# Unexpected Positive Cultures During Aseptic Hip and Knee Revision Arthroplasty: Substantial Discrepancies in Laboratory Analyses

**DOI:** 10.3390/antibiotics14040372

**Published:** 2025-04-03

**Authors:** Marius Ludwig, Michael Fuchs, Olivia Trappe, Moritz Oltmanns, Heiko Reichel, Tobias Freitag

**Affiliations:** Department of Orthopedic Surgery, University of Ulm, Oberer Eselsberg 45, 89081 Ulm, Germany

**Keywords:** periprosthetic joint infection, pathogen, contamination, microbiology, aseptic revision surgery, microbial diagnostics

## Abstract

**Background:** Microbial analysis of tissue samples represents an important diagnostic tool in the course of revision total joint arthroplasty. Currently, unexpected positive intraoperative cultures are commonly observed during presumed aseptic revision surgery and evoke a degree of uncertainty among physicians. To date, it is unclear if there are deviations in pathogen detection between certified laboratories. **Methods:** Tissue samples of sixty consecutive patients undergoing presumably aseptic total hip and knee revision surgery were sent to two different internationally certified accredited laboratories and tested for any microbial growth as well as pathogen differentiation. **Results:** Each laboratory analyzed 300 samples. Laboratory 1 observed an unexpected positive culture rate of 16.7%; laboratory 2 indicated that 18.3% of all processed specimens showed pathogen growth. In comparison, a consistent microbial evaluation was only present in one patient. The kappa correlation coefficient showed a poor correlation between the two laboratories in all evaluated categories. Coagulase-negative staphylococci represented the most common pathogens of laboratory 1, while laboratory 2 predominantly observed cutibacterium acnes species. Within a mean follow-up period of 17.6 ± 18.6 months (range: 0–63 months), there was no revision due to periprosthetic joint infection. **Conclusions:** Unexpected positive culture results during presumed aseptic revision surgery remain a significant clinical challenge. This study is the first of its kind to evaluate the convergence of laboratory findings in the context of aseptic revision surgery. Our results suggest that even established and certified laboratories show substantial discrepancies. Thus, a careful interpretation of unexpected bacterial cultures after revision surgery is mandatory. Given the uncertainty inherent in laboratory findings, a precise clinical and histopathological evaluation of this patient cohort should be ensured.

## 1. Introduction

Intraoperative tissue sample acquisition and consecutive microbiological analysis are important diagnostic steps in total hip and knee revision surgery. These investigations should be performed routinely to confirm or exclude periprosthetic joint infection (PJI), and represent essential cornerstones of current classification criteria [1,2]. Besides PJI, the most common reasons for revision total hip and knee arthroplasty are aseptic loosening, wear, instability, dislocation and periprosthetic fracture [3,4,5]. It has been speculated that some presumed aseptic failures following revision may be due to undiagnosed PJI [6,7,8]. Specifically, in cases with aseptic loosening, the underlying cause of failure might be low-grade infection. Thus, a meticulous intraoperative microbial evaluation during revision surgery is indicated. However, unexpected positive intraoperative cultures (UPICs) are commonly encountered during aseptic revision surgery of total hip (THA) and knee arthroplasties (TKA). In this context, current studies reported UPIC rates of 8.3–26.1% [7,8,9,10,11]. Recently, Vargas-Reverón et al. retrospectively evaluated 420 cases of presumed aseptic total knee or hip revision surgery. In that study, the authors observed a UPIC rate of 24.2% [12]. However, there are still contradictory data regarding the clinical implication and postoperative management of these unsuspected conditions. While some studies state that pathogen occurrence does not impair implant survival, others observe higher revision rates in the presence of unexpected positive bacterial cultures [6,7,10,12]. Moreover, contaminations during sample acquisition, transport or sample processing represent an additional cause of UPICs. If present, the latter pose a significant clinical challenge, as treatment for infection and aseptic loosening differ substantially. In addition to these challenges, there is a considerable paucity in the literature with regard to the validity and comparability of laboratory analyses. This raises the question of whether different laboratories yield comparable results with regard to pathogen detection and bacterial species classification. To date, no study is focusing on the convergence of microbial results subject to different laboratory analyses. Consequently, we hypothesized that laboratories of the same type would achieve divergent results with regard to pathogen detection rate and bacterial species classification. The main aim of this study was to evaluate UPIC findings in aseptic revision hip and knee revision surgeries and to compare their occurrence between two internationally tested and calibrated laboratories.

## 2. Results

### 2.1. Demographics

A total of 60 patients were included in this study. The mean age at time of operation was 75.4 ± 9.3 years (range: 51.9–93.9 years). Twenty-nine patients (48.3%) were male. Revision total knee arthroplasty was performed in 22 cases (27.7%), whereas 38 patients (63.3%) underwent revision THA procedures. The reasons for revision surgery were as follows: 1. aseptic loosening, 25 (41.6%); 2. instability, 7 (11.7%); 3. wear, 16 (28.3%); 4. periprosthetic fracture, 10 (16.7%); 5. component malpositioning, 1 (1.7%).

### 2.2. Laboratory Results

A total of 600 samples were analyzed. Of the 300 specimens sent to laboratory 1, 11 samples (3.7%) showed unexpected positive cultures, while 17 out of 300 samples sent to laboratory 2 displayed bacterial findings (5.7%).

In laboratory 1, the observed positive results were found in 10 patients, resulting in a UPIC rate of 16.7%. Microbial evaluation in laboratory 2 revealed that 11 patients (18.3%) had positive culture results. A merged analysis of both institutions revealed that UPICs were detected in 18 out of 60 patients (30%). In three cases (5%), both laboratory 1 and laboratory 2 displayed positive intraoperative cultures. However, consistent microbial findings were observed in only one patient (patient 4, Table 1). The kappa correlation coefficient indicated a poor correlation between the two laboratories with respect to all UPIC findings (k = 0.162, *p* = 0.207).

After postoperative application of periprosthetic joint infection guidelines according to the European Bone and Joint Infection Society (EBJIS), 4 of 10 cases in laboratory 1 and 3 of 11 cases in laboratory 2 were diagnosed as periprosthetic infections by implication of the respective microbiological results. Among these, one patient had tissue analyses revealing the growth of *Staphylococcus epidermidis* in both laboratories (patient 4, Table 1). The other cases involved discrepant assessments between the laboratories (patients 6, 9, 11, 12 and 14, Table 1). Of the six patients meeting the infection criteria, two were treated with antibiotics (patient 4: 6 weeks Vancomycin (intravenous) + Rifampicin (oral) + 4 weeks Linezolid (oral) + Rifampicin (oral); patient 9: 3 weeks Ampicillin/Sulbactam (intravenous) + Rifampicin (oral) + 9 weeks Levofloxacine (oral) + Rifampicin (oral)). For three patients (patients 6, 12 and 14), no antibiotic treatment was given as the clinical course, as the histopathology and sterile samples from the other laboratory indicated a contamination of the respective specimen. One patient (patient 11) died from heart failure due to pulmonary embolism in the early days after surgery before antibiotic treatment.

The kappa correlation coefficient further underscored substantial interrater disagreement with regard to the assessment of periprosthetic infection (k = −0.667, *p* = 0.083). Six of ten cases in laboratory 1 and eight of eleven in laboratory 2 were classified as contaminations. Two cases had positive samples in both laboratories even though the detected bacteria differed (patients 16 and 18). The kappa correlation coefficient indicated a high and statistically significant interrater disagreement concerning sample contamination (k = −0.667, *p* = 0.014).

When distinguishing between hip and knee revisions, we found a comparable rate of unexpected positive cultures in hip revisions (12/38 patients, 31.6%) and knee revisions (6/22 patients, 27.3%). With respect to bacterial strains, coagulase-negative staphylococci represented the predominant pathogen findings of laboratory 1. For laboratory 2, cutibacterium acnes was the most frequently detected microorganism (Table 2).

Within a mean follow-up period of 17.6 ± 18.6 months (range: 0–63 months), three re-revision surgeries were performed in our patient cohort of 60 individuals due to periprosthetic fracture (*n* = 2) and instability (*n* = 1). There was no further revision due to periprosthetic infection. Microbiological sample analysis of the respective re-revision surgeries showed no bacterial growth.

## 3. Discussion

Intraoperative sample acquisition and subsequent microbial analysis are a diagnostic mainstay in the course of revision arthroplasty. Current studies indicate that at least three and ideally five intraoperative periprosthetic tissue samples should undergo aerobic and anaerobic culture. As stated by the Infectious Diseases Society of America (IDSA) and the European Bone and Joint Infection Society (EBJIS), a total of five intraoperative samples appear to be the optimal compromise between sensitivity and specificity [14,15,16]. In the setting of presumed aseptic revision surgery, unsuspected positive bacterial cultures represent a considerable clinical challenge. In recent years, several studies have attempted to determine the clinical implication of these unexpected findings regarding implant survival, revision rates and the respective clinical management of UPICs [6,7,10,12]. Surprisingly, no study has yet drawn attention to potential deviations and heterogeneities of conducted analyses with respect to laboratory sample work-up. As reported previously, the latter is vulnerable to contamination during sample handling, transport and laboratory procedures [17]. As such, any potential error during sample processing could result in a UPIC and thus implicate false-positive findings in data interpretation, leading to an inherent dilemma regarding the postoperative management of these patients.

The main finding of this study is that there were substantial discrepancies between two different internationally tested and calibrated laboratories concerning UPIC detection and pathogen classification. Interestingly, the displayed results differed significantly. Neither for retrospectively declared contaminations nor for cases in which PJI was diagnosed afterwards could we find any accordance of the conducted microbial analysis (contamination, k = −0.667, *p* = 0.014; PJI, k = −0.667, *p* = 0.083). Consequently, we have to share concerns with regard to the convergence of displayed analysis. The prevalence of UPICs during presumed aseptic revision arthroplasties of the hip and knee is comparable to previously reported rates. In their systematic review, Purudappa et al. evaluated ten studies and reported that UPICs were present in 379 (10.5%) of 3605 patients undergoing revision for presumed aseptic total hip or knee arthroplasty. The authors noted a degree of interstudy heterogeneity, as the UPIC prevalence across the analyzed studies ranged from 4% to 38% [9]. This may be attributable to different microbiological sample culture techniques or divergent approaches in terms of preoperative antibiotic treatment. Vargas-Reverón et al. retrospectively evaluated 420 cases with presumed aseptic total hip or knee revision surgery and reported an overall UPIC incidence of 24.2% [12]. In our study, the prevalence of UPICs during presumed aseptic revision THA and TKA was 16.7% (laboratory 1) and 18.3% (laboratory 2). The overall UPIC rate in our study was 30%, which can be explained by the fact that only one case had congruent sample culture results between the analyzing laboratories (patient 4). Despite the combined higher overall rate when merging both analyses, our results are similar to reported values in the literature. Concerning bacterial species classification, the findings of this study are consistent with previous reports showing that UPICs are mainly caused by low-virulent microorganisms [11,18,19]. The most common causative microorganisms in our study were *Cutibacterium* (*C*.) *acnes* and coagulase-negative staphylococcus species. A study by Saleh et al. analyzed 155 UPICs (10%) from 1540 revision THAs and TKAs for aseptic loosening. The authors reported that 67% of infections were caused by coagulase-negative staphylococci and *C. acnes* [19]. Wu et al. stated that the vast majority (79.6%) of UPIC findings in a series of 691 revision TKAs were attributable to c. acnes and coagulase-negative staphylococcus species [11]. Focusing on 1196 aseptic THA revisions, Neufeld et al. observed similar results among 110 UPIC results. In their study, coagulase-negative staphylococci and C. acnes also accounted for the majority of positive bacterial cultures [18]. The clinical implication of unexpected positive bacterial cultures remains an issue of constant debate. In their study, Vargas-Reverón et al. reported that 19.5% of patients had a single UPIC, and 4.7% had either ≥ 2 UPICs for the same microorganism or 1 UPIC for a virulent microorganism. Overall, UPICs were not significantly associated with an increased risk of re-revision at a 5-year follow-up [12]. Neufeld et al. evaluated 110 UPICs from 1196 aseptic revision hip arthroplasties and found that no patient with a single UPIC developed PJI caused by the UPIC-identified organism. The authors concluded that a single UPIC does not necessitate antibiotic treatment [18]. In contrast, Wu et al. reported a higher re-revision rate due to concomitant PJI in the presence of ≥2 UPICs for the same microorganism at the time of initial aseptic TKA revision surgery [11]. There are some limitations of this study that need to be addressed when interpreting the observed findings. First, the analysis included a limited number of revision surgeries due to the single-center study design. Sonication of removed components, biomarker-based intraoperative tests such as synovial alpha-defensin, or advanced techniques such as next-generation sequencing were not performed routinely. Additionally, the possibility of microbial contamination during sample collection cannot be completely excluded. However, it has to be mentioned that all samples were obtained under strict sterile conditions by the performing surgeon due to a standardized clinical protocol. Moreover, given the significant differences in the spectrum of microorganisms identified between the laboratories, it is more likely that the observed discrepancies resulted from contaminations during sample processing rather than specimen collection. UPICs remain a significant clinical challenge that is commonly encountered during presumed aseptic revision arthroplasty. This is the first study to evaluate the convergence of laboratory findings subject to tissue sample analyses in the context of aseptic revision surgery. It has to be clearly stated that even established and internationally accredited laboratories show considerable discrepancies. Given the fact that microbial analyses of tissue cultures are highly susceptible to contamination, it is plausible that the observed differences were primarily due to unintended bacterial colonization during sample handling or laboratory processing. This issue implicates a challenge in data interpretation and subsequent postoperative patient management, creating a tremendous dilemma for physicians and clinical microbiologists. Therefore, we recommend cautious interpretation of unexpected bacterial cultures following revision surgery. As laboratory findings exhibited greater uncertainties than expected, a meticulous clinical and histopathological evaluation of this patient cohort is highly recommended.

## 4. Materials and Methods

### 4.1. Study Design

Between 1 August 2021 and 3 August 2022, consecutive patients aged 18 years or older who met the inclusion criteria of undergoing aseptic total hip or knee revision arthroplasty were prospectively enrolled. This study was approved by the local ethics committee (registration number: FSta 40/20). Informed consent was obtained before participation. Preoperatively, all patients underwent a comprehensive clinical examination, systemic blood sampling, radiographic imaging and a joint aspiration. In cases with unclear radiological examination, computed tomography imaging was performed to confirm implant loosening. EBJIS guidelines were used to classify revision surgeries as aseptic [1]. Patients under preoperative antibiotic treatment or any immunosuppressive drugs were excluded from this study.

### 4.2. Revision Surgery and Sample Collection

Revision surgeries were performed by 3 senior high-volume surgeons. Operating rooms were certified according to DIN EN ISO 14644-3 [20] requirements and had laminar airflow systems. Before surgery, a single-shot antibiotic prophylaxis (2nd generation cephalosporins) was administered intravenously. Following a standardized clinical protocol, 5 samples for microbiological analysis and 5 specimens for histological analysis were taken during revision surgery. Histological samples were collected from the prosthesis–bone interface. These periprosthetic membranes were classified according to Morawietz and Krenn [13], distinguishing between four different types. Type I indicates a periprosthetic membrane of the abrasion-induced type characterized by macrophages and giant cells with evidence of prosthesis wear. Type II, the periprosthetic membrane of the infectious type, is dominated by inflammatory tissue with neutrophile granulocytes, edema, fibroblasts and vascular proliferation. In type III, a combination of the aforementioned types is present. The periprosthetic membrane in type IV is indifferent with no aspects of type I or type II present and a high content of collagen tissue.

Each sample was placed in a separate sterile tube without culture medium or saline. In line with the study design and to enable a comparative analysis between laboratories, a double set of tissue samples (*n* = 10) was collected. Thus, each laboratory received 5 tissue samples for subsequent microbiological processing.

### 4.3. Sample Processing

Collected samples were analyzed by 2 different, accredited laboratories, both certified according to DIN EN ISO 15189 standards [21]. All specimens were processed following a standardized microbiological protocol [22]. Samples were cultured in enrichment broths (laboratory 1: Thioglycollate broth, Thermo Fisher Scientific Inc, Waltham, MA, USA; laboratory 2: Thioglycollate + Brain Heart Infusion broth, Thermo Fisher Scientific Inc., Waltham, MA USA) as well as on 5 agar plates (laboratory 1: Schaedler Agar, Thermo Fisher Scientific Inc., Waltham, MA, USA; CHROMagar^TM^ Candida, CHROMagar, Saint-Denis, France; Columbia CNA Agar, MacConkey Agar, Columbia Agar, bioMérieux, Marcy l’Etoile, France; laboratory 2: MacConkey Agar, bioMérieux, Marcy l’Etoile, France; Caso Agar, Chocolate Agar, Schaedler KV Agar, Schaedler Agar, Thermo Fisher Scientific Inc., Waltham, MA, USA) and incubated for 10 (laboratory 2) and 14 days (laboratory 1) at 35°.

In both laboratories, identification and susceptibility testing of isolated microorganisms was performed using an automatic bacteriological analyzer (ViteK 2, bioMérieux, Marcy l’Etoile, France).

### 4.4. Interpretation of Positive Bacterial Results

Cultures were considered positive if a high-virulent organism grew in ≥1 specimen of synovial fluid, periprosthetic tissue or sonication (*Staphylococcus aureus*, *Streptococcus* spp., *Candida* spp.) or a low-virulent organism grew in ≥2 specimens (coagulase-negative staphylococci, *Enterococci*, *Cutibacterium* spp., and other bacteria of the skin microbiome). A single positive culture of a low-virulent organism without further evidence of PJI (histopathology, clinical features) was defined as contamination.

### 4.5. Statistical Analysis

Statistical analysis was performed using SPSS software (IBM SPSS Statistics for Windows, Version 29.0. Armonk, NY, USA: IBM Corp.). The kappa correlation coefficient was employed to determine interrater agreement between the two laboratories. Unless otherwise specified, values are means ± 1 standard deviation. The level of significance was set at *p* = 0.05.

## Figures and Tables

**Table 1:** UPIC findings, postoperative histological assessments, and synovial fluid analyses of sterile preoperative joint aspirations.

Patient No.	Sex	Age (y)	Revision TKA/THA	Reason for Revision	Follow-Up (m)	Re-Revision	Pathogen Spectrum Laboratory 1	Pathogen Spectrum Laboratory 2	Preoperative Joint Aspiration		Histology
									WBC Count	PMN (%)	M and K Type
1	m	60.8	TKA	wear	59	no	-	*C. acnes* (1, s)	1800	12	1
2	f	58.2	THA	loosening	63	no	*S. capitis* (1, e)	-	n.a.	n.a.	1
3	m	89.5	THA	loosening	0 ^a^	no	-	*Corynebacterium* spp. (1, e)	3500	24	1
4	f	77.3	THA	fracture	2	no	*S. epidermidis* (1, s)	*S. epidermidis* (3, s), *E. coli* (1, e)	13,100 ^b^	75	-
5	f	77.6	THA	loosening	1	no	*S. epidermidis* (1, s)	-	n.a.	n.a.	4
6	m	82.3	TKA	loosening	15	no	-	*C. acnes* (2, e)	2200	10	1
7	f	85.0	THA	instability	0	no	*S. auricularis* (1, e)	-	2500	43	1
8	f	77.3	THA	fracture	28	no	-	*S. epidermidis* (1, e)	-	-	4
9	f	83.0	THA	loosening	1 ^a^	no	*S. capitis* (1, s)	-	n.a.	n.a.	3
10	m	86.2	TKA	fracture	20	yes	*Kocuria kristinae* (1, e)	-	14,350 ^b^	46	1
11	m	76.4	TKA	loosening	0 ^a^	no	-	*C. acnes* (2, e)	200	6	1
12	m	87.8	THA	wear	35	no	*E. coli* (1, e) *Enterococcus* spp. (1, s)	-	4300	46	1
13	f	76.8	TKA	wear	12	no	-	*Paenibacillus* spp. (1, e)	1200	12	4
14	f	75.4	TKA	loosening	22	no	*K. pneumoniae* (1, a)	-	1750	17	1
15	m	81.6	THA	wear	6	no	-	*C. acnes* (1, s)	n.a.	n.a.	4
16	m	66.8	THA	wear	20	no	*Kocuria rosea* (1, e)	*C. acnes* (1, s) *Paenebacillus* spp. (1, e)	5600 ^b^	55	1
17	m	73.4	THA	wear	8	no	-	*Micrococcus luteus* (1, e)	n.a.	n.a.	4
18	m	93.9	THA	fracture	24	no	*Streptococcus* spp. (1, e)	*Acinetobacter* spp. (1, e)	-	-	4

no.: number, y: years, m: months, WBC: white blood cell, PMN: polymorphonuclear leukocyte percentage, C.: *Cutibacterium*, S.: *Staphylococcus*, spp.: species, E.: *Escherichia*, K.: *Klebsiella*, ^a^: deceased due to heart failure, ^b^: cell count caused by fracture or wear, n.a.: cell count not available due to insufficient amount of joint fluid aspiration. Numbers in brackets indicate the frequency of each positive pathogen finding among the five tissue sample cultures per laboratory. e: single colonial growth after enrichment; s: single to moderate colonial growth without enrichment; a: abundant colonial growth without enrichment. Histological evaluation was undertaken according to Morawietz and Krenn (M and K) classification [13].

**Table 2:** Pathogen spectrum of UPIC.

Laboratory 1			Laboratory 2		
Coagulase-negative Staphylococci hereof:		5	*Cutibacterium acnes*		7
*S. capitis*	2				
*S. epidermidis*	2				
*S. auricularis*	1				
*Streptococcus sanguinis*		1	Coagulase-negative Staphylococci hereof:		4
			*S. epidermidis*	4	
*Enterococcus* spp.		1	*Paenibacillus* spp.		2
*Klebsiella pneumoniae*		1	*Escherichia coli*		1
*Kocuria kristinae*		1	*Corynebacterium*		1
*Kocuria rosea*		1	*Micrococcus luteus*		1
*Escherichia coli*		1	*Acinetobacter* spp.		1

Numbers indicate the frequency of each positive pathogen finding. S.: *Staphylococcus*, spp.: species.

## Data Availability

Original contributions presented in this study are included in the article. Further inquiries can be directed to the corresponding author. Raw data supporting the conclusions of this article will be made available by the authors on request.

## Abbreviations

The following abbreviations are used in this manuscript:

EBJIS	European Bone and Joint Infection Society;
IDSA	Infectious Diseases Society of America;
PJI	periprosthetic joint infection;
PMN	polymorphonuclear leukocyte percentage;
Spp.	species;
THA	total hip arthroplasty;
TKA	total knee arthroplasty;
UPIC	unexpected positive intraoperative cultures;
WBC	white blood cell.
